# Evolution of 3D Cultures: Toward Tailored Preclinical Models

**DOI:** 10.3390/cancers15020515

**Published:** 2023-01-14

**Authors:** Roberto Benelli, Maria Raffaella Zocchi, Alessandro Poggi

**Affiliations:** 1 SSD Oncologia Molecolare e Angiogenesi, IRCCS Ospedale Policlinico San Martino, 16132 Genova, Italy; 2 Divisione di Immunologia, Trapianti e Malattie Infettive, IRCCS Istituto Scientifico San Raffaele, 20132 Milano, Italy

## 1. Introduction

The identification and validation of simple, reliable and reproducible three dimensional (3D) in vitro culture systems represent a major challenge in the field of anticancer drug development.

Indeed, the study of the molecular mechanisms underlying a tumor’s patient-specific development, antitumor immunity and response to therapy turned out to be very expensive and only partially satisfactory in animal models [1,2]. Humanized murine models and patient-derived tumor xenografts (PDXs) have been extensively used, but not always with success. In particular, the different cellular and extracellular matrix components of the human tumor microenvironment (TME) are not easily reproducible in animals. Human tumor cells injected in mice do not meet the same micro- (cell and matrix component) and macroenvironment (vascular, lymphatic and nervous systems) in which the original tumor mass developed [2,3].

The idea of replacing animal models has long been thought unrealistic. Nevertheless, interest has been raised among scientists in facing the problem, in addition to the widespread awareness that breeding of experimental animals is expensive and has an environmental impact. Along this line, the 3R (Replacement, Reduction and Refinement) principle, first described in 1959 by William Russel and Rex Burch to minimize the pain and distress of research animals while maintaining scientific integrity, was taken up and developed.

Thus, the search for different 3D culture systems has been encouraged by the EU Reference Laboratories (EURL-ECVAM), and in the last ten years, a series of new systems for the selection of anticancer drugs was approved and validated [1,2,4]. Tumor cell spheroids, patient-derived organoids and repopulated scaffolds have been designed and applied to the study of the biological behavior of tumors, the antitumor immune response and the development of anticancer drugs [5,6].

Each of these systems has advantages and limitations that have been only partially resolved. Recent scientific works, briefly presented in the second section of this editorial, have proposed interesting solutions that could potentially lead to a breakthrough in the development of animal-free preclinical models.

## 2. From Spheroids to Organoids: Advantages and Drawbacks

Both established cell lines and primary isolated tumor cells can form spheroids when cultured in ultra-low adherent plastic plates or in hanging drops containing aggregated tumor cells that form a microsphere [5,6,7,8]. Spheroids can be composed of cancer cells only (homotypic) or two or more (heterotypic) cell types, including fibroblasts, thus adding to the 3D system at least one component of the complex network of cells present in the TME [2,5]. In addition, spheroids can be obtained using primary cancer cells isolated from patients; this allows the setting of autologous 3D systems that are useful to study the interaction between cancer and immunocompetent cells and the effects of different anticancer drugs [5,6]. The main advantages of the spheroid 3D cultures are feasibility, low cost, reproducibility and high-throughput screening.

As an alternative, patient-derived organoids, generated from tumors and composed of tumor cells at different stages of differentiation, have been extensively used to reproduce a reliable in vitro preclinical system [6,9]. Tumor organoids can be obtained from a specimen containing tumor epithelial and nonepithelial components in an air–liquid interface culture system, or by culturing epithelial cells embedded in a matrix, where they can expand due to the activity of mitogenic components and factors acting on epithelial stem cells [6,9,10,11].

Organoids share many advantages with spheroids, such as large sampling, reproducible analyses and the autologous setting of tumor cell–immune cell interactions; a limitation is represented by the heterogeneous yield from patient to patient that makes their potential employment unpredictable.

Both tumor spheroids and tumor organoids have constraints in mimicking the physiological conditions of the host, as they do not display the complexity of the original tissue. Indeed, spheroids are mainly aggregates of tumor cells only and organoids gradually lose, during culturing, the accessory cells of the TME, reducing them to a structure of epithelial tissue at different stages of differentiation [11]. In particular, tumor-associated fibroblasts and myofibroblasts are progressively lost in the subsequent in vitro passages. These features make the two models feasible and reliable in short-term experiments, such as drug screening and toxicity evaluations.

## 3. Tissue Architecture and Structure: Synthetic Scaffolds or Decellularized Matrices?

The absence of tissue architecture and structure in a 3D culture system turned out to represent a major drawback: indeed, alterations to the composition and framework of the extracellular matrix (ECM) is known to shape the behavior of different solid neoplasms [12,13]. Uncontrolled modifications to the three-dimensional (3D) structure of the tissue induce remodeling and, eventually, improve cancer progression and dissemination [14,15]. Recently, a direct correlation has been proposed between biochemical and ultrastructural ECM modifications, which influence mechanoelastic features, cancer invasiveness, the severity of the disease and even the response to therapy [15,16].

Synthetic scaffolds, such as microfibrillar collagen Ultrafoam sponges, which are suitable for being repopulated by cultured tumor cells, have been proposed to solve the problem [1,13]. Unfortunately, in many instances, cancer cells do not fit with such structures, as they are not able to reshape them [17,18]. As an alternative, gelatin-based matrices that allow for consistent remodeling by co-cultured cells have been successfully used for both solid and hematological tumor 3D models [17,18]. Nevertheless, these biomaterials also have some disadvantages, such as being difficult to embed and manipulate for immunohistochemical studies, mainly due to their intrinsic hydration.

Another solution is represented by decellularized matrices that are prepared by different protocols based on serial enzymatic treatments of bioptic or surgical samples, which leads to an ECM scaffold that maintains the original architecture, is free of cells and is suitable for a certain degree of remodeling by cells newly cultured on them. Two main disadvantages of these 3D systems are their heterogeneity, in terms of size dimension and number of achievable samples, and the difficult standardization procedure.

## 4. Seed and Soil Hypothesis

Two recent papers from the same group point to a 3D model able to mimic either the organ-specific or the metastatic microenvironment. They start from the observation that patient cancer cells, especially metastatic cells, can be considered as a “seed” that is barely able to be implanted into a microenvironment, the “soil”, which is very different from that encountered in vivo. In particular, the ECM composition differs from one organ to another and the metastatic niche is continuously modified by cancer cells themselves. In turn, the ECM can shape not only the local spreading of tumor cells, but also the metastatic process [19,20].

In the first study, the authors describe a protocol for the decellularization of healthy colon (HC) and colorectal cancer (CRC) tissue in order to obtain matrices with a conserved composition and ultrastructure [19]. The protocol differs from others previously described [13,18], as it combines the use of sodium deoxycholate and DNase, resulting in acellular scaffolds that support cell survival and are actively repopulated. In addition, the tissue architecture and structure were preserved and deeply analyzed by immunohistochemistry and scanning electron microscopy. Drug absorption was tested by microscopy, exploiting the autofluorescence of doxorubicin detectable in the inner part of the scaffolds.

Gelatin scaffolds that fulfil all these requirements have been described by others [17,18]; nevertheless, the decellularization proposed by this group offers the advantage of a “soil” with biochemical and biophysical characteristics that is superimposable to the original tissue and organ. The system, validated in zebrafish xenotransplants and tested for 5-fluorouracile (5-FU) and FOLFIRI (folinic acid plus irinotecan, followed by 5-FU) efficacy, is presented as a reliable in vitro 3D preclinical model that can allow for the study of primary and metastatic cancers, differentiating their responses to therapy [19].

In the second study, the authors developed an ex vivo 3D model of colorectal cancer liver metastasis (CRLM) and matched CRC by applying the decellularization protocol to patient-derived liver and colorectal specimens [20]. Again, the demonstration of a structure and architecture recapitulating the original tissues is provided, underlying the reticular collagen distribution crucial for metastatic cell migration, in keeping with previous reports [13,18].

Along this line, seeded tumor cells migrate better into CRLM and CRC scaffolds than in HC or in healthy liver (HL) scaffolds, conceivably due to their different architecture and structure. Indeed, the liver stroma is a network of sinusoidal microvessels, mostly composed of reticular collagen, and a discontinuous endothelium cell lining, which is easily accessible to metastatic cells. In addition, tumor cells cultured on CRML decellularized matrices down regulated the expression of E-cadherin, moving toward an epithelia-to-mesenchyme transition that favors metastasization [20].

The paper offers a deep molecular analysis that highlights the upregulation in CRLM scaffolds of genes involved in demethylation, deacetylation, the response to stress and hypoxia, and provides further evidence for the organ and tissue specificity of this 3D culture model. Finally, as the most challenging finding, it turns out that the CRLM scaffold composition reduces the sensitivity of cancer cells to 5-FU and FOLFIRI, which is at variance with CRC or HL scaffolds, providing the proof of principle for an organ-specific 3D preclinical model. The low proliferation rate of CRC cells within the 3D scaffolds is claimed by the authors as the current preclinical gold standard for assessing drug efficacy [20].

## 5. Conclusions

It is now widely recognized that the TME, especially the ECM composition and structure, is strictly entwined with tumor fate; nevertheless, neither animal nor 3D in vitro models have, thus far, fulfilled all the requirements for reliable, reproducible and unexpensive preclinical settings.

In particular, although organotypic, patient-derived decellularized matrices repopulated with tumor cells seem to be representative of the real tissue(s) where cancers develop, there are still some limits to this 3D culture model. First, there is an absence of stromal, endothelial and accessory cells actually recapitulating the complex TME or metastatic microenvironment. Second, the yield and the homogeneity of samples available for drug testing and immune response assessment in an autologous setting are not ideal. Third, there is a lack of dynamic conditions to completely reproduce the in vivo landscape. Certainly, these drawbacks will be overcome in the next future due to the growing progresses in biotechnology and imaging.
